# Low agreement among patients and clinicians about urgency and safety to wait for assessment in primary care after hours medical care: results of cross-sectional matched surveys

**DOI:** 10.1186/s12913-023-09399-3

**Published:** 2023-05-02

**Authors:** Katelyn Barnes, Caitlin Arpel, Kirsty Douglas

**Affiliations:** 1 Academic Unit of General Practice, Office of Professional Leadership and Education, ACT Health Directorate, Canberra, Australia; 2 School of Medicine and Psychology, College of Health and Medicine, Australian National University, Canberra, Australia

**Keywords:** Health services research, Health systems, Health seeking behaviour, Primary care, Emergency department, Afterhours care, Survey

## Abstract

**Background:**

Discordance between patient and clinician perceived urgency may drive “inappropriate” presentations to after-hours medical services. This paper investigates the level of agreement between patient and clinicians’ perceptions of urgency and safety to wait for an assessment at after-hours primary care services in the ACT.

**Methods:**

Cross-sectional survey voluntarily completed by patients and then clinicians at after-hours medical services in May/June, 2019. Agreement between patients and clinicians is measured by Fleiss kappa. Agreement is presented overall, within specific categories of urgency and safety to wait, and by after-hours service type.

**Results:**

888 matched records were available from the dataset. Overall inter-observer agreement between patients and clinicians on the urgency of presentations was slight (Fleiss kappa = 0.166; 95% CI 0.117–0.215, p < 0.001). Agreement within specific ratings of urgency ranged from very poor to fair. Overall inter-rater agreement on how long it would be safe to wait for assessment was fair (Fleiss kappa = 0.209; 95% CI 0.165–0.253, p < 0.001). Agreement within specific ratings ranged from poor to fair. By site type, agreement between patients and clinicians on urgency ranged from not significant to fair and agreement for safety to wait ranged from very poor to slight. Agreement on urgency of issue was more often reported among patients attending their usual health service or seeing their usual clinician compared to patients attending an unfamiliar health service or clinician (χ^2^(1) = 7.283, p = 0.007 and χ^2^(1) = 16.268, p < 0.001, respectively).

**Conclusions:**

Low levels of agreement between patients and clinicians on perceived urgency and safety to wait for issues to be assessed indicate potential inefficiency in primary care use after-hours. Agreement on urgency of issues was more common among patients attending a familiar health service or familiar clinician. Improving health literacy, particularly health system literacy, and supporting continuity of care may help to support patients to engage with the most appropriate level of care at the most appropriate time.

## Introduction

Accessible, cost effective, and efficient after-hours medical care in Australia continues to be a focus and challenge for policy makers and health system managers. Currently, a hybrid model of Emergency Departments (ED) and community-based medical services exist outside of routine working hours (9am – 5pm) and during weekends. Emergency Department (ED) presentations are high cost and increasing, with an estimated 40% labelled as “potentially avoidable” [1, 2]. As such, Australian government initiatives plan to reduce “potentially avoidable” ED presentations, particularly in the after-hours periods, with the current focus on GP Urgent Care Centres [1–6].

The most common patient reasons for presenting to after-hours medical services are perceived need and sense of urgency [7–12]. However, clinicians rarely assess presentations as urgent as patients do [7, 9, 10, 12, 13]. Current understanding of Australian patient and clinician sense of urgency are only from ED settings; with only one international study set in community-based after-hours services [12]. While after-hours primary care in Australia manages a mixture of urgent and non-urgent presentations [11, 14–16]; studies report unmatched proportions of patient perceived urgency and clinician reported appropriateness of presentations [7, 9, 10, 12], with only two papers considering case-by-case basis [12, 13]. As such discordance between patient perceived and clinician perceived urgency of a presenting problem may underpin the concept of “potentially avoidable” presentations to after-hours medical services. Matched assessments of perceived urgency between patients and clinicians may help understand current after-hours Australian primary care services, and inform strategies to enhance efficiencies in after-hours access[7, 13].

This paper investigates the level of agreement between matched patient and clinician perceptions of urgency and safety to wait for an assessment for medical encounters at after-hours primary care services in Australia. Findings can inform after-hours models of care and public health messages to direct patients to the most appropriate service at the most appropriate time.

## Method

This study examines data collected from a broader project that explored presentations in after-hours primary care [11, 14]. A cross-sectional survey voluntarily completed by practitioners and patients was undertaken in May/June, 2019 from 6pm on Thursday to 8am Monday; covering one weeknight and weekend. Data was not collected Friday 8am-6pm. The project received ethical approval through the ACT Health Human Research Ethics Committee and Calvary Public Hospital Bruce Human Research Ethics Committee (2019/LRE/00003 and CPHB-HREC 10-2019). All methods were conducted in accordance with the declaration of Helsinki.

### Participant pool

Participants and recruitment processes have been reported [11, 14]. Briefly, potential participants included (i) any person presenting to a study site during the study period, without obvious barriers to consent, and (ii) any clinician providing care at any medical service in the ACT, open after 6pm Thursday or Friday for at least two hours, or anytime Saturday or Sunday. Data was collected from 50 General Practices with varied billing practices, three Medical Deputising Services (MDS) staffed by GPs generally incurring a co- payment; three Nurse led Walk in Centres (WICs) offering services free at point-of-care; and two public EDs, with services free at point-of-care. Patients can choose to attend any of the services, though are dependent on opening hours and availability of clinicians [11]. GP services are site specific with some providing “walk-in” options, and most open until 6pm weekdays and at least 4 h on Saturday morning; WICs are a “walk in” service only, open 7am-10pm every day; MDS is an appointment and “walk in” service available after 6pm weekdays and anytime on weekends; and ED is a “walk in” service open at all times.

Participants were invited by a research assistant in the waiting room. Patients < 18 years of age, were included if a carer or parent was able to provide informed consent. Practitioners from the EDs were unable to participate. As such, matched data from ED was unavailable. Not all practitioners consented to take part. Patients seeing non-consenting practitioners were still eligible to take part in the patient survey, but could not contribute matched patient data. Unmatched patient data are reported elsewhere [11].

### Questionnaire tools

The research team developed the patient questionnaire, which was informed by literature review and consultation with health care clinicians and consumers. The questionnaire contained 26 questions including a mixture of multiple choice, Likert scale and short answer questions. Patients completed the survey prior to their consultation. Patients could give their health care clinician, at their discretion, a unique code number and consent card to assist data matching.

The research team developed the clinician questionnaire, which was modelled on the Bettering the Evaluation of Care and Health (BEACH) survey [17]. The clinician recorded the patient’s unique code to support paired data analysis. Clinicians completed the questionnaire during or after the patient consultation, with access to their usual diagnostic resources. Details of both patient and clinician survey tools have been previously published [11, 14].

This paper focusses on survey questions: “How urgent is this issue?” and “How long could this issue wait to be safely assessed?” from both patient and clinician questionnaires.

For urgency, patients and clinicians were asked, “How urgent is the problem you are having assessed today?” with answer options including: “An urgent issue (e.g. *I have a new pain or my asthma is no longer under control*)”, ‘A non-urgent issue (e.g. *I need a repeat script or a routine pap smear*); or ‘Uncertain about urgency’. For safety to wait, patients and clinicians were asked, ‘How long do you think it would be safe to wait to have this problem assessed?’, For analysis, data was condensed into: ‘Immediately (within 1 hour)’, ‘Within 12 hours’, ‘Within 12–48 hours’ and ‘Within the next week or so’.

### Data analysis

Only matched patient and clinician data were used in this analysis. Frequencies, percentages, median and IQR (where required) were used to describe patient characteristics with a matched record. Matched record patient characteristics were compared to ACT population data from 2019 [18], and the whole patient dataset [11] using Chi square goodness of fit.

Cross tabulations were generated for patient versus clinician perceptions of safety to wait and urgency. Percentages within tables were calculated as the proportion of patient responses that were agreed upon by the clinician. Proportion of patient responses was considered the most appropriate as this paper focusses on patient perceptions as a driving factor in presentation. For example, if 100 patients reported their problem as ‘Urgent’, and only 20 of their respective clinicians’ responses matched, the agreement is reported as 20%. Total columns for all patient responses and clinician responses are reported.

Fleiss’ kappa was used to indicate inter-rater agreement [19] for overall ratings of urgency and safety to wait, as well as within specific categories (e.g. agreement within urgent, uncertain, and non-urgent ratings) and is reported alongside 95% Confidence Intervals and p values. Unlike Cohen’s kappa which relies on the same two raters for all cases, Fleiss kappa accounts for each case being rated by a different clinician and patient pair [20]. Table 1 indicates interpretation of kappa scores [20]. Two new bivariate outcomes were calculated to identify when patients and clinicians rated urgency or safety the same (agreement) or different. Pearson chi square was used to identify if agreement was associated with using a familiar health service and with seeing a familiar clinician.

**Table 1:** Interpretation of Fleiss’ Kappa

Value of k	Strength of Agreement
<-0.01	Very poor
0	Only by chance
0–0.20	Slight
0.21–0.40	Fair
0.41–0.60	Moderate
0.61–0.80	Substantial
0.81–1.00	Almost perfect

## Results

Study population including sites and total patient population have been previously reported [11, 14]. Matched patient questionnaires and consultation records were identified for 888 participants (31.2% of all presentations, and 53.5% of all study participants). Table 2 describes the matched patient population.

**Table 2:** Patient demographic and characteristic descriptors

Demographic or characteristic	n (%)
*Sex*	
Male	368 (41.5)
Female	515 (58.1)
Prefer not to say	5 (0.5)
*Age*	
0–9 10–19 20–60 60–69 70+ Not reported Median years (IQR)	146 (16.4) 105 (11.8) 485 (54.6) 57 (6.4) 47 (5.3) 48 (5.4) 32 (31.3)
*Identify as Aboriginal or Torres Strait Islander*	
Yes	14 (1.6)
No	864 (97.3)
Prefer not to say	10 (1.2)
*Language other than English at home*	
English	662 (74.5)
Other	218 (24.5)
Not reported	8 (0.9)
*Is this your usual health service?*	
No	341 (38.4)
Yes	517 (58.2)
Prefer not to say	30 (3.4)
*Are you seeing your usual doctor today?*	
No	672 (75.7)
Yes	177 (19.9)
Prefer not to say	39 (4.4)

Patients with matched records differ from the general ACT population, with over representation of females (51.3% vs. 50.6% p < 0.001) and underrepresentation of males (47.0% vs. 49.4%, p < 0.001) [18]. Patients with matched records did not differ significantly from the total study sample with respect to demographic or characteristic variables [11].

Of 315 clinicians working during the study period, 86 participated (27.3%). Most clinicians were General Practitioners (n = 56, 65.1%), of whom six were registrars. Other clinicians included Registered Nurses (n = 25, 29%) and Nurse Practitioners (n = 5, 5.8%).

Matched ratings of presentation urgency were available for 828 participants. Matched and total patient and clinician ratings of urgency are shown in Table 3, by site and overall.

**Table 3:** Matched patient and clinician ratings of the urgency of after-hours presentation by site and overall

	Site		Clinician rating, n(%)^a^			*Patients total, n(%)*	*Fleiss Kappa (95%CI)*
			**Non-urgent**	**Uncertain**	**Urgent**		
**Patient rating, n (%)**	GP	Non-urgent	173 (79.1)	15 (6.7)	32 (14.2)	*220 (0.511)*	0.317(0.223–0.441)^**^
		Uncertain	70 (53.4)	17 (12.9)	44 (33.6)	*131 (0.297)*	-0.009 (-0.103-0.085)
		Urgent	29 (34.5)	12 (14.2)	43 (51.1)	*84 (0.191)*	0.248 (0.154–0.342) ^**^
		*Site subtotal*	*272*	*44*	*119*	*435*	0.207 (0.138–0.275)^**^
	MDS	Non-urgent	10 (45.5)	3 (13.6)	9 (40.9)	*22 (0.134)*	0.082 (-0.072-0.235)
		Uncertain	25 (30.4)	10 (12.1)	47 (57.3)	*82 (0.503)*	-0.130 (-0.284-0.023)
		Urgent	14 (23.7)	2 (3.4)	43 (72.9)	*59 (0.361)*	0.116 (-0.038-0.269)
		*Site subtotal*	*49*	*15*	*99*	*163*	0.025 (-0.86-0.136)
	WIC	Non-urgent	52 (73.2)	6 (8.5)	13 (18.3)	*71*	0.195 (-0.066-0.325) ^*^
		Uncertain	57 (47.1)	16 (13.2)	48 (39.7)	*121*	-0.146 (-0.276 - -0.017) ^*^
		Urgent	14 (37.8)	3 (11.1)	20 (54.1)	*37*	0.110 (-0.020-0.239)
		*Site subtotal*	*123*	*25*	*81*	*229*	0.056 (-0.036-0.149)
	Overall	Non-urgent	235 (75.1)^k^	24 (7.7)	54 (17.3)	313 (37.8)	0.302 (0.234–0.370) ^**^
		Uncertain	152 (45.4)	43 (12.8)^l^	140 (41.8)	335 (40.6)	-0.064 (-0.132-0.004)
		Urgent	57 (31.7)	17 (9.4)	106 (58.9)^m^	180 (21.7)	0.214 (0.146–0.282) ^**^
		*Clinicians total, n (%)*	*444 (53.6)*	*84 (10.1)*	*300 (36.2)*	*828 (100)*	0.166 (0.117–0.215) ^**^

^a^ Percentage of patient ratings that were rated, respectively, as Non-urgent / uncertain / Urgent by the clinician.

^*^ p < 0.05

^**^ p < 0.01

Overall, the majority of patients were uncertain of the urgency for their presentation (n = 335; 40.6%), while the majority of clinicians rated presentations as non-urgent (n = 444; 53.6%). Of the patient rated urgent presentations (n = 180), clinicians rated 58.9% as urgent, 31.7% as non-urgent and 9.4% as uncertain. Both patients and clinicians agreed on urgency of presentations in 46.4% of cases (n = 384). Overall inter-observer agreement between patients and clinicians on the urgency of presentations was slight. Agreement within specific ratings of urgency ranged from very poor to fair.

Agreement on the urgency of presentations between patients and GPs within GP services was slight. Patients and GP agreement within specific categories of urgency ranged from fair to very poor. Agreement between patients and Locum doctors through the CALMS services was slight, though not significant. Patient and locum GP agreement within specific categories of urgency ranged from very poor to slight. Agreement on the urgency of presentations between patients and Nurses within WIC services was slight, though not significant. Patients and Nurse agreement within specific categories ranged from very poor to slight. Patients attending their usual health service, or seeing their usual health professional, more often reported agreement with their clinician on urgency of their issue compared to patients not attending their usual health service or not seeing their usual health clinician (χ^2^(1) = 7.283, p = 0.007 and χ^2^(1) = 16.268, p < 0.001, respectively) .

Matched ratings of how long it would be safe to wait for assessment was available for 845 participants. Matched and total patient and clinician ratings for safety to wait are shown in Table 4, by site and overall.

**Table 4:** Matched patient and clinician ratings of how long it would be safe for a presentation to wait to be assessed for after-hours presentations by site and overall

	Site		Clinician rating, n(%)^a^				*Patients totals, n(%)*	*Fleiss Kappa (95%CI)*
			**Immediate**	**Within 12 h**	**In 12–48 h**	**In a week or so**		
**Patient rating, n (%)**	GP	Immediately	5 (12.5)	5 (12.5)	21 (55.0)	8 (20.0)	*39*	0.088 (0.005–0.182)
		Within 12 h	5 (7.3)	21 (30.4)	32 (47.8)	10 (14.5)	*68*	0.258 (0.165–0.351)^**^
		In 12–48 h	15 (6.3)	19 (8.4)	146 (61.5)	57 (23.8)	*237*	0.159 (0.066–0.252) ^**^
		In a week or so	1 (1.0)	5 (4.8)	43 (41.3)	54 (52.9)	*103*	0.278 (0.186–0.371) ^**^
		*Site subtotal*	*26*	*50*	*242*	*129*	*447*	0.206 (0.145–0.268) ^**^
	MDS	Immediately	0 (0.0)	9 (64.3)	5 (35.7)	0 (0.0)	*14*	-0.062(-0.216 -0.092)
		Within 12 h	3 (4.0)	32 (43.2)	37 (50.0)	2 (2.8)	*74*	0.184 (0.030–0.338) ^*^
		In 12–48 h	2 (2.9)	11 (15.9)	54 (78.3)	2 (2.9)	*69*	0.246 (0.092–0.399) ^**^
		In a week or so	0 (0.0)	1 (20.0)	4 (80.0)	0 (0.0)	*5*	-0.029 (-0.183-0.125)
		*Site subtotal*	*5*	*53*	*100*	*4*	*162*	0.177 (0.053–0.301) ^**^
	WIC	Immediately	1 (2.4)	22 (52.4)	19 (45.2)	0 (0.0)	*42*	-0.063 (-0.193-0.066)
		Within 12 h	2 (2.5)	39 (48.8)	34 (42.5)	5 (6.2)	*80*	0.132 (0.003–0.262) ^*^
		In 12–48 h	1 (1.0)	29 (30.5)	58 (61.1)	7 (7.4)	*95*	0.140 (0.011–0.270) ^*^
		In a week or so	0 (0.0)	1 (8.3)	8 (66.7)	3 (25.0)	*12*	0.173 (0.044–0.303) ^**^
		*Site subtotal*	*4*	*91*	*119*	*15*	*229*	0.111; (0.019–0.203) ^*^
	Overall	**Immediate**	6 (6.3)	36 (37.9)	45 (47.4)	8 (8.4)	*95 (11.3)*	0.016 (-0.052-0.084)
		**Within 12 h**	10 (4.5)	92 (41.3)	104 (46.6)	17 (7.6)	*223 (26.6)*	0.256 (-0.189-0.324) ^**^
		**In 12–48 h**	18 (4.5)	59 (14.7)	258 (64.3)	66 (16.5)	*401 (47.9)*	0.172 (0.104–0.240) ^**^
		**In the next week or so**	1 (0.8)	7 (5.8)	55 (45.8)	57 (47.5)	*120 (14.3)*	0.316 (0.248–0.384) ^**^
		**Clinician totals**, ***n (%)***	*35 (4.2)*	*194 (23.1)*	*462 (55.1)*	*148 (17.6)*	*839 (100)*	0.209 (0.165–0.253) ^**^

^a^ Percentage of patient ratings that were rated, respectively, as Immediately / Within 12 h / In 12–48 h by the clinician.

^*^ p < 0.05

^**^ p < 0.01

The majority of patients rated their presentations as safe to wait for assessment > 12-hours (n = 521; 62.1%). Clinicians rated most presentations as being safe to wait > 12-hours (n = 615, 72%). Both patients and clinicians reported the same ratings in 49.2% of cases (n = 413). Overall inter-rater agreement on how long it would be safe to wait for assessment was fair. Agreement within specific ratings ranged from poor to fair.

Agreement between patients and GPs within GP services about safety to wait for assessment was fair. Patient and GP agreement within specific categories of safety to wait ranged from slight to fair. Agreement between patients and locum GPs within MDS services about safety to wait for assessment was slight. Patient and locum GP agreement within specific categories of safety to wait ranged from very poor and not significant to fair. Agreement between patients and Nurses within WIC services about safety to wait for assessment was slight. Patient and Nurse agreement within specific categories ranged from very poor and not significant to slight. No observed correlation occurred between agreement on safety to wait and patients attending a familiar health service or familiar health professional.

## Discussion

This paper reports low levels of agreement between patients and clinicians on perceived urgency and safety to wait for assessment, indicating potential inefficiencies in primary care use after-hours. Low agreement is not unexpected as patients and clinicians approach medical problems from different perspectives [7]. Identifying where disagreements occur may help highlight strategies to improve efficiency in primary care use and access after-hours.

Slight inter-rater agreement was observed between patients and clinicians about the urgency of presentations. Low levels of overall agreement may be due to the very poor agreement within the “uncertain” category. Clinicians less often rated the urgency of presentations as uncertain compared to patients, which is expected given clinicians training and role in assessing and managing health conditions. Low levels of overall agreement may also occur from both over- and under-estimations of urgency from patients compared to their clinicians. One-third of patients (31.5%) who felt their problem was urgent were rated as non-urgent by clinicians, and nearly a fifth of patients (17%) who believed their problem was non-urgent were rated as urgent by the clinicians. Patient under-estimation may occur when patients present with a benign symptom but the consultation unearths more complex issues (e.g., a patient presents for a repeat pain relief script to manage their “usual headache” but the clinician discovers malignant hypertension). Patient over-estimation may occur where a patient presents with what they fear is life-threatening, but proves to be benign (e.g., a patient presents with abdominal pain fearing acute appendicitis that the clinician finds to be constipation). The frequency of both over- and under-estimation of urgency by patients highlights the complexity of patient health-seeking behaviours. While strategies aimed at improving individual health literacy and self-management may support patients to select the most appropriate health service at the most appropriate time [21, 22]; it could also have an unintended consequence of increasing patients’ general health concerns and therefore drive increased presentations of the “worried well” [22]. Clearly, multiple strategies are needed to support patients to select the most appropriate service at the most appropriate time.

Fair inter-relator agreement was observed between patients and clinicians on how long presentations could safely wait to be seen. Agreement was poorest in the group where patients perceived the need to be seen immediately, with only 6% of clinicians making the same assessment. Low proportions of over- and under-estimation of safety to wait were promising andmay indicate that the patient judgments on when to be seen were mostly safe.

Most presentations in the after-hours period could have safely waited until usual hours to be seen. Patients and clinicians agreed in 37.5% of cases that the assessment could wait > 12 h. If this 37.5% of patients had waited > 12 h to be seen, many would have moved from presenting after-hours to presenting during usual hours. Compared to care during usual hours, after-hours care has higher staffing costs, fewer staff available, and minimal access to ancillary services such as pathology and radiology [3, 5, 23]. While we recognise that presentations are also driven by patient preference and prioritisation around other life commitments [11]; this proportion of non-urgent patients being seen in the after-hours period is significant, and likely reduces the availability of after-hours primary care for urgent “GP type” presentations. Urgent “GP type” presentations after-hours may instead present to other services, such as Emergency Departments.

Greater agreement between patients and clinicians on urgency and safety to wait for assessment was reported within GP compared to other services, and greater agreement between patients and familiar clinicians. Greater agreement in the GP setting may be underpinned by the relationship-based and ongoing care provided by GPs, where the clinician better understands a patients psycho-social context alongside the presenting issue and can make a more contextually informed assessment of urgency for each patient [24]. In contrast, the models of care provided by WICs and MDS are much more transactional and the clinicians are unlikely to have supporting knowledge of the patient’s social situation or their prior responses to illness episodes. Therefore the clinicians in WIC and MDS can only judge urgency from a medical perspective, without the benefit of greater contextual background. These results highlight the importance of clinician-patient relationships and continuity of care for patient assessment and understanding. However, comparing agreement across services should note the differences in purpose and problems managed:: WICs are advertised as providing care for minor non-urgent issues and often manage wounds; GPs offer usual care on weekends and often manage chronic conditions, infections, and preventive health issues; MDS provide GP-type care that cannot wait for the availability of a usual GP and often manage infections [14]. Findings from this paper aligns with previous studies that show the majority of patients are using services as advertised, though some patients remain unclear on where to present [11, 14].

Strategies to improve efficiencies in after-hours medical services in Australia are currently focussed on diverting patients away from ED [1–6]. However, this study shows that more effort is needed to direct patients in selecting the most appropriate alternate ED service for their needs, at the most appropriate time. Improved health literacy could be supported by increased advertising via public health and individual services, to both members of the public and health service counterparts [5]. Advertisement should focus on availability and scope of services, out-of-pocket cost of accessing services after-hours and during usual hours, as well as the benefits of continuity of care. Advertisements could encourage seeing a familiar clinician whenever possible, and promote accessing services during usual hours whenever possible, even if there may be a short delay in care for non-urgent issues. More broadly, patients should be supported to access care during usual hours when possible, such as encouraged use of sick leave for healthcare appointments [11].

This paper has several strengths and limitations. This is the first study to use matched patient and clinician data from primary care in the after-hours periods, providing a good indication of patient health-seeking behaviours. While number of matched records available was high, the data is representative of a third of presentations to primary care during the study period, and only half of participants in the wider study. High dropout rate from the wider study to matched questionnaires (47%), may partly be due to patients not sharing their study details with their clinician, and partly due to multiple clinicians choosing not to take part. High dropout may indicate discomfort with collecting personal medical data, and may mean that patients with common and non-serious issues were more likely to take part. As such, our results may underestimate the perceived urgency from both patient and clinician viewpoints. However, patients may have experienced a desirability bias where they over reported the urgency of their presentation, which may have increased observed discordance with clinician ratings. Anonymous surveys and data collection being separate from reception or point of care were attempts to mitigate desirability bias. The study sample had a slight overrepresentation of females compared to the ACT population and may not be reflective of the broader Australian population; however, this is reflective of the gender balance of people interacting with the health system [25]. Furthermore, the ACT health system and population may have different structures (e.g., Nurse led WIC) and health seeking behaviours compared with other health districts. However this data may be useful to inform possible interventions to improve efficiencies in after-hours care, and may provide useful comparative data. Finally, data were collected before the COVID-19 pandemic, and it is unclear if and how COVID-19 and related health system changes have altered patient health-seeking behaviours.

Recent efforts to improve health outcomes whilst reducing the burden of care in the formal health system have focussed on the need to improve patients’ health literacy and self-management skills. This study indicates that improving health system literacy and supporting continuity of care may be important components of this overall strategy to help promote utilisation of the appropriate level of care at the appropriate time. Ideally, patients will develop an understanding of not only what their health problem may be and how to manage it, but also where within the health system their problem can be managed effectively and efficiently, as well as how and when to engage with each service.

## Data Availability

The data that support this study cannot be shared publicly due to ethical or privacy reasons. De-identified data may be shared upon reasonable request to the corresponding author and approval from the original institutional ethics committee.
